# Changes of the Thioredoxin System, Glutathione Peroxidase Activity and Total Antioxidant Capacity in Rat Brain Cortex During Acute Liver Failure: Modulation by l-histidine

**DOI:** 10.1007/s11064-014-1417-9

**Published:** 2014-08-27

**Authors:** Joanna Ruszkiewicz, Jan Albrecht

**Affiliations:** Department of Neurotoxicology, Mossakowski Medical Research Centre, Polish Academy of Sciences, 02-106 Warsaw, Poland

**Keywords:** Thioredoxin, Glutathione peroxidase, Hepatic encephalopathy, Histidine, Oxidative/nitrosative stress, Total antioxidant capacity

## Abstract

Glutathione and thioredoxin are complementary antioxidants in the protection of mammalian tissues against oxidative–nitrosative stress (ONS), and ONS is a principal cause of symptoms of hepatic encephalopathy (HE) associated with acute liver failure (ALF). We compared the activities of the thioredoxin system components: thioredoxin (Trx), thioredoxin reductase (TrxR) and the expression of the thioredoxin-interacting protein, and of the key glutathione metabolizing enzyme, glutathione peroxidase (GPx) in the cerebral cortex of rats with ALF induced by thioacetamide (TAA). ALF increased the Trx and TrxR activity without affecting Trip protein expression, but decreased GPx activity in the brains of TAA-treated rats. The total antioxidant capacity (TAC) of the brain was increased by ALF suggesting that upregulation of the thioredoxin may act towards compensating impaired protection by the glutathione system. Intraperitoneal administration of l-histidine (His), an amino acid that was earlier reported to prevent acute liver failure-induced mitochondrial impairment and brain edema, abrogated most of the acute liver failure-induced changes of both antioxidant systems, and significantly increased TAC of both the control and ALF-affected brain. These observations provide further support for the concept of that His has a potential to serve as a therapeutic antioxidant in HE. Most of the enzyme activity changes evoked by His or ALF were not well correlated with alterations in their expression at the mRNA level, suggesting complex translational or posttranslational mechanisms of their modulation, which deserve further investigations.

## Introduction

Acute liver failure (ALF) causes a spectrum of pathogenic events in the brain culminating in astrocytic swelling and brain edema, whereby accumulation of high concentrations of ammonium ions (ammonia) in blood and brain appear to be the main causative factor [1–4]. Evidence from different studies suggest that oxidative/nitrosative stress (ONS) plays a key role in the pathogenesis of hepatic encephalopathy (HE) associated with ALF. Increased manifestations of events related to ONS, e.g.: superoxide accumulation, lipid peroxidation, protein nitration and nitrosylation, nucleic acid oxidation, and changes in antioxidant systems were found in the brains of ALF in patients and animals, and in in vitro models ([5–9], recently reviewed in [10]).

Glutathione (GSH), the most abundant low molecular antioxidant in brain has been shown to be involved in the response to ALF, but the evidence has not been unambiguously conclusive. *In vitro* studies revealed that ammonia leads to a decrease of GSH content in cultured neurons [11] but to an increase in cultured astrocytes [12, 13], which was associated with increased uptake of its precursor amino acid, cystine [13]. In the in vivo setting, thioacetamide (TAA)-induced ALF or simple acute hyperammonemia (HA) resulted in increased extracellular GSH accumulation and degradation in rat brain [14]. On the other hand, a significant decrease was observed in the brain mitochondria during ALF in rats [15]. Depending on the HA or ALF model employed, the brain activity of the key GSH-metabolizing enzyme, glutathione peroxidase (GPx) was noted to either go up [16] or down [17, 18].

In addition to the GSH system, the antioxidative responses of mammalian tissues, including the brain, engage one other thiol-reducing system, the thioredoxin (Trx) system [19–22]. Thioredoxins (Trxs) reduce disulfides in proteins and their regeneration takes place through thiol reduction by the flavo- and selenoprotein–thioredoxin reductase (TrxR) with NADPH. Mammalian tissues express two main Trx systems, the cytosolic couple: Trx1/TrxR1 and the mitochondrial couple: Trx2/TrxR2. The activity of the Trx system is also controlled by an endogenous factor, thioredoxin-interacting protein (Trxip) [23, 24]. All the components of the Trx system are known to be present in the central nervous system (CNS) tissues (references in [21, 22]). By analogy to GSH [12–14], it may be speculated that Trx is likewise an astroglia-derived neuroprotectant. TrxR and GSH-metabolizing GPx are selenium (Se)-containing antioxidants. There is a high priority for Se supply in the brain and even minimal alteration of Se-containing enzymes activity is correlated with neurodegenerative diseases associated with ONS, such as Alzheimer’s (AD) or Parkinson’s disease (references in [19, 21, 22]). While selenoprotein alterations in HE-affected brain have not been directly studied, changes in Se-transporting proteins have been reported in blood of HE patients [25]. Bearing in mind the above data, in the present study we investigated whether ALF induces changes in Se-dependent Trx/TrxR system and GPx activity, and what is the relation between the two systems in the response to ALF.

l-histidine (His) counteracts brain edema in ALF [26] by a mechanism involving reduction of ammonia-induced mitochondrial permeability transition and astrocytic swelling in vitro [27], and correction of GSH imbalance in ALF-affected brain [15]. It thus appeared of interest to see whether and in what degree His would interfere with the effects of ALF on GPx and Trx system. Finally, to evaluate the effectiveness of the sum of responses of the two systems to ALF and/or His, we measured the total antioxidant capacity of the tissue under these conditions.

## Experimental Procedure

### ALF Model

ALF was induced in adult male Sprague–Dawley rats (220–280 g) by three intraperitoneal (i.p.) injections of thioacetamide (TAA), 300 mg/kg body weight, at 24 h intervals [28]. Typical symptoms of acute HE are observed in this model, including elevated blood and brain ammonia levels and brain edema associated with increased brain Gln, the magnitude of the changes resembling severe stages of HE in humans ([26, 28], and references therein). His at a dose of 100 mg/kg was injected i.p. 2 h before each TAA injection; His injection does not affect ammonia level in the brain [26]. Controls received equivalent volume of saline. Animals were sacrificed 24 h after the last administration. Tissue from brain cortex was homogenized in a buffer appropriate to each protocol or frozen at −80 °C.

### GPx Activity

GPx activity was measured as previously described in [12], the procedure being based on the original method of [29], with minor modifications. Fresh tissue was homogenized (1/8; w/v) in a buffer: 50 mM Tris (pH 7.6), 0.25 mM EDTA, 0.5 mM DTT, centrifuged (10,000×*g*, 5 min, 4 °C) and supernatant was used to analyze enzyme activity and protein level. The reaction mixture: 100 mM Tris (pH 7.6), 0.5 mM EDTA, 1 mM DTT, 1 mM GSH, 0.2 mM NADPH and 0.4 unit/ml glutathione reductase was added to approx. 40 μg of protein extract. Addition of 0.2 mM *tert*-butyl hydroperoxide (TBHP) (final volume 0.2 ml) initiated the reaction and the change in absorbency of NADPH (extinction coefficient 6.22 mM^−1^ cm^−1^) was measured at 340 nm for 5 min, at 0.5 min interval, at 25 °C. Simultaneously a nonspecific reduction of NADPH was determined in each probe without TBHP. The results are presented as nmol/(min mg of protein).

### TrxR Activity

TrxR activity was measured using a Thioredoxin Reductase Assay Kit (Sigma Aldrich) according to the manufacturer’s protocol for 96 well plate assay. Tissue was homogenized (1/4; w/v), centrifuged (10,000×*g*, 5 min, 4 °C) and supernatant was used for analysis of enzyme activity and protein level. Approx. 100 μg of protein extract was used in a reaction mixture, addition of 5,5′-dithiobis-(2-nitrobenzoic acid) (DTNB) solution initiated the reaction and the change in absorbency caused by 2-nitro-5-thiobenzoic acid (TNB; extinction coefficient 14.15 mM^−1^ cm^−1^) production was measured at 415 nm for 2 min, at 10 s interval (at 25 °C). The results are presented as nmol/(min mg of protein).

### Trx Activity

Trx was determined according to the method of [30]. Fresh tissue was homogenized (1/8; w/v) in a buffer: 50 mM Tris (pH 7.6), 5 mM EDTA and protease inhibitor cocktail, centrifuged (10,000×*g*, 5 min, 4 °C) and supernatant was used for analysis of enzyme activity and protein level. Samples (approx. 40 μg of protein) were incubated 20 min in 37 °C in a reaction mixture: 85 mM HEPES (pH 7.6), 2.5 mM EDTA, 0.3 mM insulin, 0.72 mM NADPH with or without 100 nM TrxR to determine non-specific reduction of insulin (final volume 0.1 ml). Insulin reduced through Trx system reacts afterwards with 1 mM DTNB (in 6 M guanidine hydrochloride, pH 8.0) added at the end of incubation (final volume 0.3 ml). Absorbance was measured at 415 nm and the results were calculated using extinction coefficient for the product of the reaction—TNB in guanidine hydrochloride (13.6 mM^−1^ cm^−1^), and presented as nmol/mg of protein [31].

### TrxR1 and Trxip Assay

TrxR1 and Trxip protein level were determined by enzyme-linked immunosorbent assay (ELISA) Kits (MyBioSource; catalog nos. MBS881784 and MBS261228). Tissue was homogenized and centrifuged, and supernatant was used for ELISA according to the manufacturer’s protocol. The results are presented as ng/mg of protein.

### Total Antioxidant Capacity

Total antioxidant capacity (TAC) was determined using an Antioxidant Assay Kit (Sigma–Aldrich, catalog no. CS0790). Fresh tissue was homogenized (1/5; w/v) in Assay Buffer, centrifuged (12,000×*g*, 15 min, 4 °C) and supernatant was used to analysis of TAC and protein level. Approx. 30 μg of protein extract was used in reaction mixture, which contained myoglobin and 2,2′-azino-bis(3-ethylbenzthiazoline-6-sulfonic acid) (ABTS). Addition of hydrogen peroxide produces ferryl myoglobin radical which oxidizes ABTS to a chromogen detected spectrophotometrically at 415 nm. Trolox™ curve was used as a standard for antioxidants’ concentration and results were calculated as nmol Trolox™/mg of protein.

### Protein Determination

The results of enzymes activity and ELISA assay were calculated on protein content, determined using BCA Protein Assay Kit (Pierce) according to manufacturer’s protocol.

### Quantitative Real Time PCR (qRT-PCR)

Total RNA was isolated from frozen rat cortex using the standard protocol for Tri reagent [32]. To remove genomic DNA, a Recombinant DNase I (rDNase I) (Ambion) was used according to the method described by a producer. Complement DNA was synthesized from 1 μg of RNA with Reverse Transcription Kit (Applied Biosystems). The level of gene expression was measured with a real-time PCR System using TaqMan Gene Expression Assay (Applied Biosystems) with 1 μl cDNA in 10 μl of reaction volume. The assay IDs were: Rn00587437_m1 (*Txn1,* Trx1), Rn00584162_g1 (*Txn2,* Trx2), Rn01503798_m1 (*Txnrd1,* TrxR1), Rn00574868 (*Txnrd2*, TrxR2), Rn00577994_g1 (*Gpx1,* Gpx1), Rn01533891_g1 (*Txnip*, Trxip), Rn00667869_m1 (*βActin*, β-actin—internal control). Change in gene expression was determined by the 2^−ΔΔCt^ method [33] and expressed as relative change to control level.

### Statistical Analysis

The results were expressed as mean ± SD. Differences among experimental groups were evaluated by a one-way ANOVA analysis of variance with post hoc Tukey’s test. *P* < 0.05 was considered statistically significant.

## Results

**Fig. 1:**
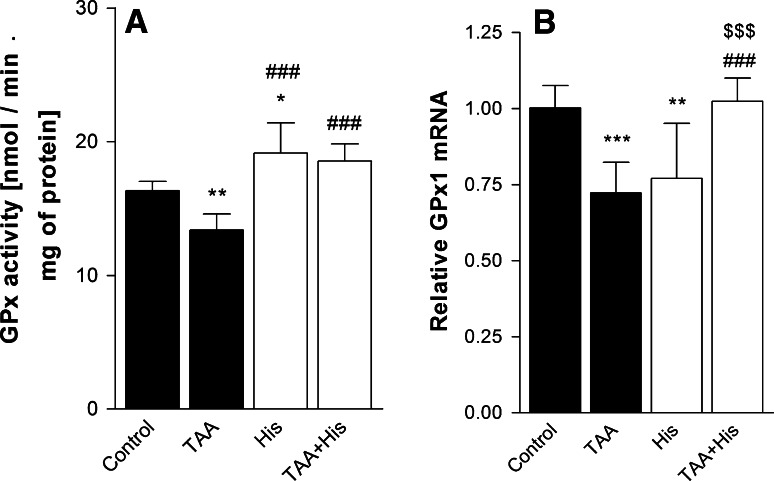
Effect of ALF in the TAA model (TAA) or/and histidine (His) administration on glutathione peroxidase (GPx) activity (**a**, n = 6) and glutathione peroxidase 1 (GPx1) mRNA level (**b**, n = 7–8) in rat brain cortex. Results are mean values ± SD. *p < 0.05, **p < 0.01, ***p < 0.001 versus Control, ^###^ *p* < 0.001 versus TAA, ^$$$^ *p* < 0.001 versus His

**Fig. 2:**
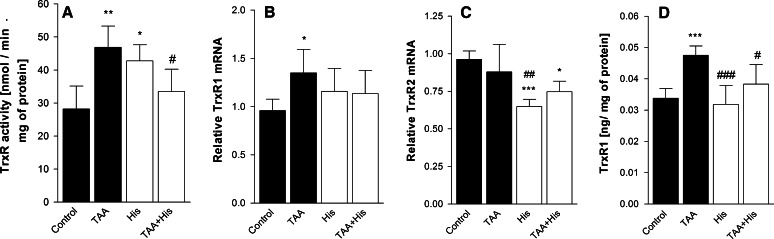
Effect of ALF in the TAA model (TAA) or/and histidine (His) administration on thioredoxin reductase (TrxR) activity (**a**, n = 4–5), thioredoxin reductase 1 (TrxR1) (**b**, n = 6), thioredoxin reductase 2 (TrxR2) (**c**, n = 6–7) mRNA level and TrxR1 protein level (**d**, n = 6) in rat brain cortex. Results are mean values ± SD. *p < 0.05, **p < 0.01, ***p < 0.001 versus Control, ^#^ *p* < 0.05, ^##^ *p* < 0.01, ^###^ *p* < 0.001 versus TAA

**Fig. 3:**
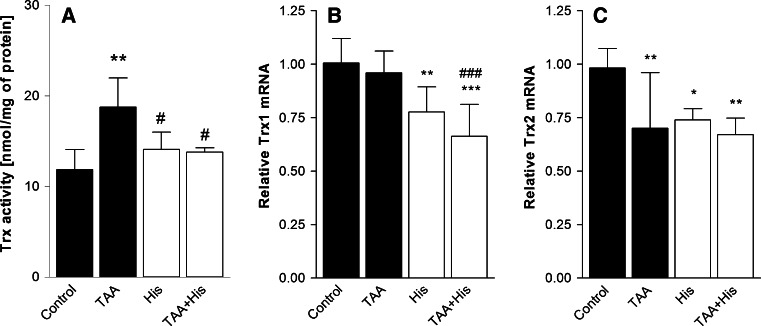
Effect of ALF in the TAA model (TAA) or/and histidine (His) administration on thioredoxin (Trx) activity (**a**, n = 4–5), and the expression of mRNAs coding Trx1 (**b**, n = 7–8) and Trx2 (**c**, n = 7–8) in rat brain cortex. Results are mean values ± SD. *p < 0.05, **p < 0.01, ***p < 0.001 versus Control, ^#^ *p* < 0.05, ^###^ *p* < 0.001 versus TAA

ALF decreased by 23 % the GPx activity (Fig. 1a) but increased by 66 % the TrxR activity (Fig. 2a), and by 65 % the Trx-reducing activity (Fig. 3a). Administration of His abrogated all the enzyme activity changes evoked by ALF (Figs. 1a, 2a, 3a). His added alone significantly increased the activities of TrxR or GPx, and tended to increase the Trx activity, albeit the increase did not reach statistical significance.

Increase of the GPx activity in the ALF animals treated or not treated with His correlated positively with the increased mRNA level of GPx1 isoform. No such a correlation occurred upon treatment with His alone, which evoked a decrease of GPx1 mRNA (Fig. 1b). ALF increased both TrxR1 mRNA level (Fig. 2b) and protein expression (Fig. 2d), which was reflected by elevation of TrxR activity (Fig. 2a). Co-administration of His abolished the effects of ALF (Fig. 2a, b, d). TrxR2 mRNA level was not changed after TAA treatment, but decreased after His administration (Fig. 2c). The mRNA coding for the cytosolic isoform of Trx, Trx1 remained unchanged comparing to control level (Fig. 3b), and the expression of mRNA coding for the mitochondrial Trx isoform—Trx2, was decreased marginally after TAA treatment (Fig. 3c). His down-regulated the expression of both Trx isoforms. Taken together, except for the coordinated response of TrxR at the level of transcription, translation and activity to ALF, other changes in the enzyme activities poorly correlated with the expression of their mRNAs.

**Fig. 4:**
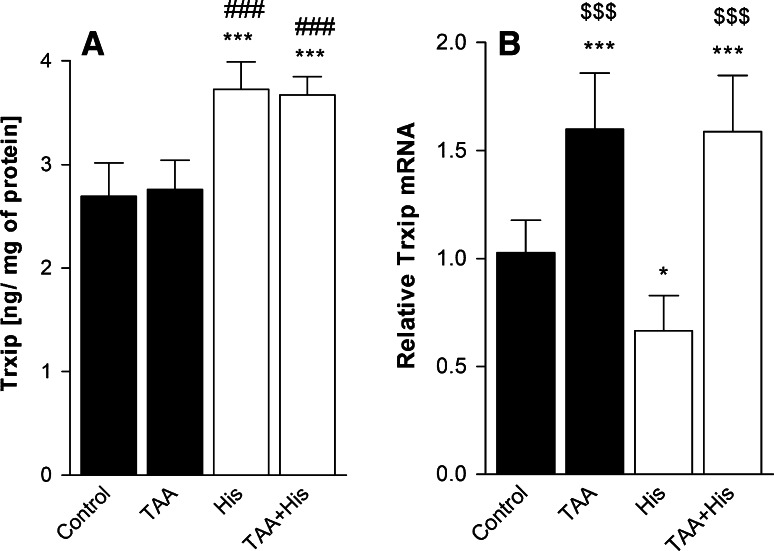
Effect of ALF in the TAA model (TAA) or/and histidine (His) administration on thioredoxin-interacting protein (Trxip) protein (**a**, n = 6–7) and mRNA level (**b**, n = 6–8) in rat brain cortex. Results are mean values ± SD. *p < 0.05, ***p < 0.001 versus Control, ^###^ *p* < 0.001 versus TAA, ^$$$^ *p* < 0.001 versus His

While ALF did not affect the expression of the thioredoxin-interacting protein (Trxip), the Trxip content was increased significantly after His treatment, both in control brains and brains derived from ALF rats (Fig. 4a). As was the case with previous Trx system components, changes in Trxip protein content did not correspond with Trxip mRNA levels, which were increased in ALF rats treated or not treated with His, but decreased upon His treatment alone (Fig. 4b).

**Fig. 5:**
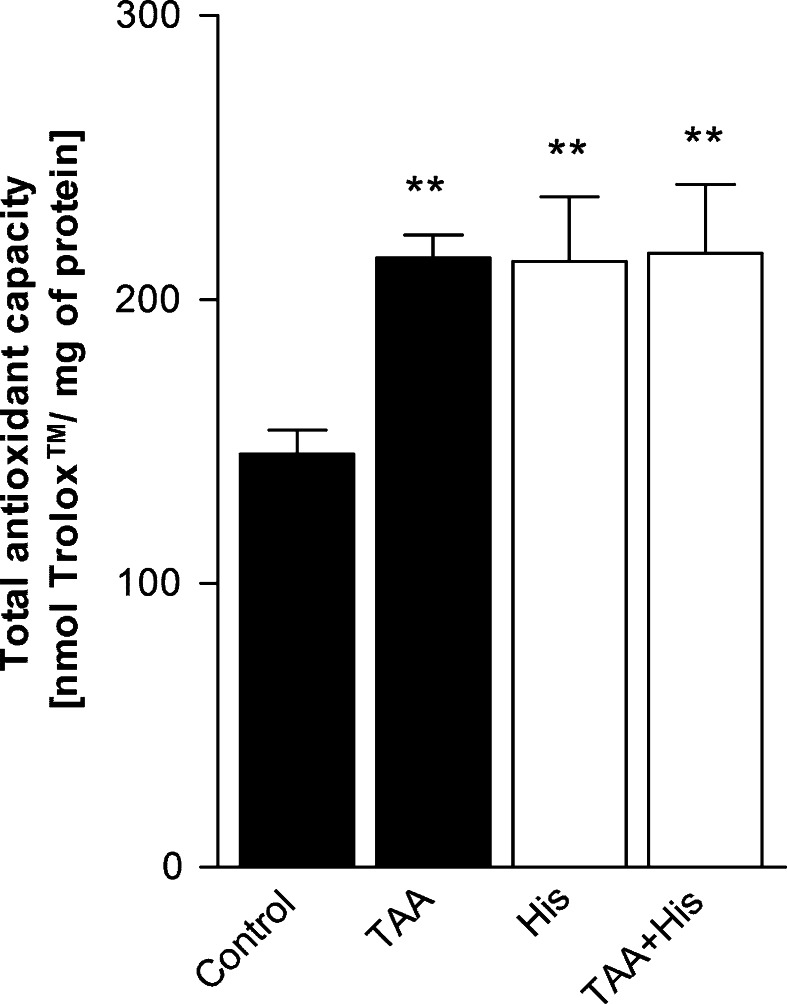
Effect of ALF in the TAA model (TAA) or/and histidine (His) administration on total antioxidant capacity in rat brain cortex. Results are mean (n = 3–4) values ± SD. **p < 0.01 versus Control

Both ALF and His treatment of control rats increased by 50 % the total antioxidant capacity (TAC) of rat brain cortical homogenates, and an increase of identical magnitude was noted in ALF rats treated with His (Fig. 5).

## Discussion

While the predominant role of the glutathione system in antioxidant defense of the brain has been established beyond doubt [34], relatively little is known about the physiological significance of the thioredoxin system in this capacity [20–22]. The present study is to our knowledge the first in the literature to address the role of the Trx/TrxR system relative to the GSH system, in the response of the brain to ALF.

ONS evoked by different factors activates various pathways leading to elevated antioxidant activity, which is often considered to be adaptive [35]. Increase in total glutathione content (GSx) in cultured astrocytes treated with ammonia, which is a HE model in vitro, is interpreted this way [12, 13]. However in the present ALF model, no changes in GSx level, GSH/GSSG ratio or glutathione reductase (GR) activity were observed in the cerebral prefrontal cortex [15]. On the top of that, mitochondrial GSH, which is considered to be a key factor in antioxidant protection, and GPx activity showed a decrease in rat brain cortex during ALF ([15] and Fig. 1a, respectively). This result corresponds to the decrease of the GPx activity previously found brain cortex of rats in the TAA model of ALF similar to that employed in the present study [17], rats with acute HA [18, 36], and in astrocytes treated with ammonium ions [12]. The antioxidant function of GPx mainly consists in reducing hydrogen peroxide through a mechanism requiring GSH as a electron donor and GR with NADPH for GSH regeneration. Decreased activity of GPx might lead to reduced elimination of hydrogen peroxide and, in consequence, increased presence of metabolites evoking ONS [34]. Since down-regulation of GPx, next to impaired mitochondrial GSH pool [15] is an Achilles’ heel in GSH-dependent antioxidant system, the results suggest that GSH may not be effective enough in coping with the ONS evoked by ALF. By contrast, both TrxR (Fig. 2a) and Trx (Fig. 3a) activities increased significantly in ALF rats. We interpret the increased Trx/TrxR system activity as a neuroprotective response to ONS in ALF. In this context, upregulation of Trx/TrxR system activity in ALF might compensate for the reduced efficiency of the GSH system, and contribute to the increase of TAC in ALF rats.

The role of the effect of ALF on the thioredoxin system is difficult to relate to the findings in other settings of brain pathology, as the available evidence concerning the Trx/TrxR response in different neurological disorders is ambiguous. Upregulation of brain Trx was demonstrated in newborn rats subjected to hyperoxia [37], and in adult mice in which brain ischemia was induced by transient middle cerebral artery occlusion [38]. In AD affected brain, TrxR activity was reported to be increased, whereas Trx level was decreased [39], rendering the overall picture inconclusive. Trx was also found decreased in astrocytes treated with an oxidative stress-inducing agent, *t*-butylhydroquinone [40]. On the other hand, the observation of elevated Trx level in astrocytes during ischemia [41], and its active release from an astroglia-derived cell line exposed to hydrogen peroxide [42], would suggest its potential role as an astroglia-derived neuroprotective factor.

To evaluate whether the enzyme activity changes observed in ALF and/or His treated rats are related to gene expression we analyzed the levels of mRNAs coding for GPx1 and the major Trx and TrxR isoforms. Decreased GPx1 mRNA level (Fig. 1b) is consistent with decreased GPx activity (Fig. 1a) in brain cortex of rats with ALF. Out of the eight different GPx isoforms present in mammalian tissues [43] we concentrated on GPx1, the first selenoprotein identified [44]. Of note in this context, GPx1 is considered to be the most vulnerable to Se deficiency among all selenoproteins—GPx1 mRNA decreases in Se deficiency in different tissues [43, 45]. The hypothesis that Se metabolism may be altered in HE was based on studies demonstrating a decrease of Se and Se-transporting selenoprotein P level in plasma of patients with cirrhosis [25], and an increase in mRNA level of two other selenoproteins (SelV and SelS) in post mortem brains with HE [46]. The results of the present study are generally consistent with the above hypothesis. More detailed studies on Se metabolism in ALF-affected brain appear warranted in this context.

Poor correlation between mRNA expression and enzyme activities for Trx and TrxR indicates the primary involvement of translational or posttranslational mechanisms in the ALF-induced changes, perhaps compounded by posttranscriptional modification of mRNA itself. Of note, RNA oxidation [5] and protein nitration and phosphorylation are the phenomena reported to accompany ONS induced in cultured astrocytes by ammonia [47]. Quite exceptionally, in the case of the TrxR1 isoform, increased TrxR activity in ALF (Fig. 2a) correlated with the increase of both the transcript level (Fig. 2b) and protein level (Fig. 2d).

To get more insight into the mechanism underlying Trx activity regulation, we decided to study the fate of the endogenous Trx inhibitor, thioredoxin-interacting protein (Trxip). Trxip which exerts its inhibitory action by binding to the active site of Trx [23], is thought to be vulnerable to different pathophysiological changes, some of which are also typical of HE. For instance, decreased glucose metabolism decreases Trxip expression [24], whereas accumulation of glutamine [48] or nitric oxide [49], or *N*-methyl-d-aspartate receptor (NMDAR) activation are known to up-regulate the expression of this protein [50]. The reasons why Trxip protein level was not affected by ALF alone, and why this occurred despite elevation of mRNA level is unclear. The discrepancies once again point to the fact that ALF may differently effect expression of one and the same factor at the transcriptional and translational level. Since Trx-Trxip binding might be disrupted as a consequence of accumulation of reactive oxygen species [23], we cannot exclude that even though Trxip protein expression is unchanged by ALF, the accumulating reactive oxygen species may impair its binding to Trx.

As outlined in the Introduction, His exerts beneficial effects on the status of ALF-affected brain and of ammonia- or glutamine-treated astrocytes in culture, by preventing mitochondrial dysfunction and ONS [26, 27]. A previous study from this laboratory demonstrated that His may render antioxidant protection by restoring brain tissue GSH [15]. The present observations that: (1) His up-regulated GPx and TrxR activity in control rats, and (2) abolished the ALF-induced changes in the Trx/TrxR system and GPx activity, further support the cytoprotective potential of this amino acid in the setting of ALF. We think that in both cases an effect of His could be a consequence of the improvement of mitochondrial function which occurs by increasing mitochondrial GSH level [15] and of the interference with Gln transport in astrocytes [27]. An alternative or additional possibility stems from the direct antioxidative potential of His, which includes reactive oxygen species scavenging or prevention of ONS generation by metal binding [51]. Studies on the effect of His on the reactive oxygen and nitrogen species in different compartments of control- and ALF-affected brain need to be carried out: such experiments are under way in this laboratory. His also up-regulated Trxip expression in both control and ALF rats as well, an observation that escapes simple interpretation. As was the case with the responses to ALF, effects of His on mRNA and protein levels are often opposite. These discrepant effects could, for instance, be related to the potent interaction of His with zinc ions [52]. Zinc is a critical regulator of activity for numerous transcription factors [53, 54] and other proteins, including TrxR [55]. ONS-related free zinc accumulation was observed in cultured astrocytes treated with ammonia [56]. Assuming that similar phenomena occur in ALF, His, through interaction with free zinc could affect gene expression and/or activity of other antioxidants. The above mentioned effects of His may render adaptive up-regulation of other antioxidant systems less critical.

The total antioxidant capacity (TAC) of the rat brain cortex was increased in rats subjected to ALF, and to His in both control and ALF rats (Fig. 5). The increase noted in ALF rats bespeaks an autoprotective response against oxidative stress. It is to be emphasized here that ALF and other forms of HE are considered to be a reversible condition only infrequently associated with astrocytic or neuronal death [57]. With regard to the interplay between the two antioxidative systems under study, the most simple interpretation is that the decreased GSH system activity is effectively compensated by the Trx system. Clearly, this attractive hypothesis deserved further detailed investigations which must also include simultaneous in-depth analysis of the fate of other cellular antioxidants contributing to TAC. As to His, it remains to be investigated by which of the multiple mechanisms attributed to this amino acid underlies its TAC-improving activity. Whatever the nature of its action, the observed effect support earlier suggestions that His has a potential to serve as a therapeutic modality in HE.

## References

[1] Clemmesen JO, Larsen FS, Kondruo JK, Hansen BA, Ott P (1999). Cerebral herniation in patients with acute liver failure is correlated with arterial ammonia concentration. Hepatology.

[2] Bernal W, Hall C, Karvellas CJ, Auzinger G, Sizer E, Wendon J (2007). Arterial ammonia and clinical risk factors for encephalopathy and intracranial hypertension in acute liver failure. Hepatology.

[3] Kato M, Sugihara J, Nakamura T, Muto Y (1989). Electron microscopic study of the blood–brain barrier in rats with brain edema and encephalopathy due to acute hepatic failure. Gastroenterol Jpn.

[4] Jalan R, Alde Damink SW, Hayes PC, Deutz NE, Lee A (2004). Pathogenesis of intracranial hypertension in acute liver failure: inflammation, ammonia and cerebral blood flow. J Hepatol.

[5] Görg B, Qvartskhava N, Keitel V, Bidmon HJ, Selbach O, Schliess F, Häussinger D (2008). Ammonia induces RNA oxidation in cultured astrocytes and brain in vivo. Hepatology.

[6] Häussinger D, Görg B, Reinehr R, Schliess F (2005). Protein tyrosine nitration in hyperammonemia and hepatic encephalopathy. Metab Brain Dis.

[7] Swapna I, Sathya Sai Kumar KV, Murthy ChR, Senthilkumaran B (2006). Membrane alterations and fluidity changes in cerebral cortex during acute ammonia intoxication. Neurotoxicology.

[8] Kosenko E, Kaminsky Y, Kaminsky A, Valencia M, Lee L, Hermenegildo C, Felipo V (1997). Superoxide production and antioxidant enzymes in ammonia intoxication in rats. Free Radic Res.

[9] Corbalán R, Chatauret N, Behrends S, Butterworth RF, Felipo V (2002). Region selective alterations of soluble guanylate cyclase content and modulation in brain of cirrhotic patients. Hepatology.

[10] Skowrońska M, Albrecht J (2013). Oxidative and nitrosative stress in ammonia neurotoxicity. Neurochem Int.

[11] Klejman A, Węgrzynowicz M, Szatmari EM, Mioduszewska B, Hetman M, Albrecht J (2005). Mechanisms of ammonia-induced cell death in rat cortical neurons: roles of NMDA receptors and glutathione. Neurochem Int.

[12] Murthy ChR, Bender AS, Dombro RS, Bai G, Norenberg MD (2000). Elevation of glutathione levels by ammonium ions in primary cultures of rat astrocytes. Neurochem Int.

[13] Węgrzynowicz M, Hilgier W, Dybel A, Oja SS, Saransaari P, Albrecht J (2007). Upregulation of cerebral cortical glutathione synthesis by ammonia in vivo and in cultured glial cells: the role of cystine uptake. Neurochem Int.

[14] Hilgier W, Węgrzynowicz M, Ruszkiewicz J, Oja SS, Saransaari P, Albrecht J (2010). Direct exposure to ammonia and hyperammonemia increase the extracellular accumulation and degradation of astroglia-derived glutathione in the rat prefrontal cortex. Toxicol Sci.

[15] Ruszkiewicz J, Fręśko I, Hilgier W, Albrecht J (2013). Decrease of glutathione content in the prefrontal cortical mitochondria of rats with acute hepatic encephalopathy: prevention by histidine. Metab Brain Dis.

[16] Kosenko E, Kaminsky Y, Lopata O, Muravyov N, Kaminsky A, Hermenegildo C, Felipo V (1998). Nitroarginie, an inhibitor of nitric oxide synthase, prevents changes in superoxide radical and antioxidant enzymes induced by ammonia intoxication. Metab Brain Dis.

[17] Reddy PV, Murthy ChR, Reddanna P (2004). Fulminant hepatic failure induced oxidative stress in nonsynaptic mitochondria of cerebral cortex in rats. Neurosci Lett.

[18] Satpute RM, Lomash V, Hariharakrishnan J, Rao P, Singh P, Gujar NL, Bhattacharya R (2012). Oxidative stress and tissue pathology caused by subacute exposure to ammonium acetate in rats and their response to treatments with alpha-ketoglutarate and N-acetyl cysteine. Toxicol Ind Health.

[19] Patenaude A, Murthy MRV, Mirault ME (2005). Emerging roles of thioredoxin cycles enzymes in the central nervous system. Cell Mol Life Sci.

[20] Masutani H, Bai J, Kim YC, Yodoi J (2004). Thioredoxin as a neurotrophic cofactor and an important regulator of neuroprotection. Mol Neurobiol.

[21] Lilling CH, Holmgren A (2007). Thioredoxin and related molecules—from biology to health and disease. Antioxid Redox Signal.

[22] Hanschmann EM, Godoy JR, Berndt C, Hudemann C, Lillig CH (2013). Thioredoxins, glutaredoxins, and peroxiredoxins—molecular mechanisms and health significance: from cofactors to antioxidants to redox signaling. Antiox Redox Signal.

[23] Zhou R, Tardivel A, Thorens B, Choi I, Tschopp J (2010). Thioredoxin-interacting protein links oxidative stress to inflammasome activation. Nat Immunol.

[24] Yu FX, Chai TF, He H, Hagen T, Lou Y (2010). Thioredoxin-interacting protein (Txnip) gene expression. Sensing oxidative phosphorylation status and glycolytic rate. J Biol Chem.

[25] Burk RF, Early DS, Hill KE, Palmer IS, Boeglin ME (1998). Plasma selenium in patients with cirrhosis. Hepatology.

[26] Rama Rao KV, Reddy PVB, Tong X, Norenberg MD (2010). Brain edema in acute liver failure. Inhibition by l-histidine. Am J Pathol.

[27] Albrecht J, Norenberg MD (2006). Glutamine: a Trojan horse in ammonia neurotoxicity. Hepatology.

[28] Hilgier W, Olson J (1994). Brain ion and amino acid contents during edema development in hepatic encephalopathy. J Neurochem.

[29] Ursini F, Maiorino M, Gregolin C (1985). The selenoenzyme phospholipid hydroperoxide glutathione peroxidase. Biochim Biophys Acta.

[30] Arner ESJ, Holmgren A (2000) Measurement of thioredoxin and thioredoxin reductase. Curr Protoc Toxicol Chapter 7, Unit 7.4 10.1002/0471140856.tx0704s05 20954152

[31] Kumar S, Holmgren A (1999). Induction of thioredoxin, thioredoxin reductase and glutaredoxin activity in mouse skin by TPA, a calcium ionophore and other tumor promoters. Carcinogenesis.

[32] Chomczynski P, Sacchi N (1987). Single-step method of RNA isolation by acid guanidinium thiocyanatephenol–chloroform extraction. Anal Biochem.

[33] Livak KJ, Schmittgen TD (2001). Analysis of relative gene expression data using real-time quantitative PCR and the 2^−ΔΔCt^ method. Methods.

[34] Dringen R, Pawlowski PG, Hirrlinger J (2005). Peroxide detoxification by brain cells. J Neurosci Res.

[35] Limón-Pacheco J, Gonsebatt ME (2009). The role of antioxidants and antioxidant-related enzymes in protective responses to environmentally induced oxidative stress. Mutat Res.

[36] Singh S, Koiri RK, Trigun SK (2008). Acute and chronic hyperammonemia modulate antioxidant enzymes differently in cerebral cortex and cerebellum. Neurochem Res.

[37] Bendix I, Weichelt U, Strasser K, Serdar M, Endesfelder S, Haefen C, Heumann R, Ehrkamp A, Felderhoff-Mueser U, Sifringer M (2012). Hyperoxia changes the balance of the thioredoxin/peroxiredoxin system in the neonatal rat brain. Brain Res.

[38] Tanaka N, Ikeda Y, Ohta Y, Deguchi K, Tian F, Shang J, Matsuura T, Abe K (2011). Expression of Keap1-Nrf2 system and antioxidant proteins in mouse brain after transient middle cerebral artery occlusion. Brain Res.

[39] Lovell MA, Xie C, Gabbita P, Markesbery WR (2000). Decreased thioredoxin and increased thioredoxin reductase levels in Alzheimer’s disease brain. Free Radic Biol Med.

[40] Eftekharpour E, Holmgren A, Juurlink BHJ (2000). Thioredoxin reductase and glutathione synthesis is upregulated by t-butylhydroquinone in cortical astrocytes but not in cortical neurons. Glia.

[41] Tomimoto H, Akiguchi I, Wakita H, Kimura J, Hori K, Yodoi J (1993). Astroglial expression of ATL-derived factor, a human thioredoxin homologue, in gerbil brain after transient global ischemia. Brain Res.

[42] Hori K, Katayama M, Sato N, Ishii K, Waga S, Yodoi J (1994). Neuroprotectection by glia cells through adult T cell leukemia-derived factor/human thioredoxin (ADF/TRX). Brain Res.

[43] Brigelius-Flohe R, Maiorino M (2013). Glutathione peroxidases. Biochim Biophys Acta.

[44] Flohé L, Günzler WA, Schock HH (1973). Glutathione peroxidase: a selenoenzyme. FEBS Lett.

[45] Sunde RA, Raines AM (2011). Selenium regulation of the selenoprotein and nonselenoprotein transcriptomes in rodents. Adv Nutr.

[46] Görg B, Bidmon HJ, Häussinger D (2013). Gene Expression profiling in the cerebral cortex of patients with cirrhosis with and without hepatic encephalopathy. Hepatology.

[47] Schliess FI, Görg B, Fischer R, Desjardins P, Bidmon HJ, Herrmann A, Butterworth RF, Zilles K, Häussinger D (2002). Ammonia induces MK-801-sensitive nitration and phosphorylation of protein tyrosine residues in rat astrocytes. FASEB J.

[48] Kaadige MR, Looper RE, Kamalanaadhan S, Donald EA (2009). Glutamine-dependent anapleurosis dictates glucose uptake and cell growth by regulating MondoA transcriptional activity. PNAS.

[49] Forrester M, Seth D, Hausladen A, Christine EE, Foster MW, Matsumoto A, Benhar M, Marshall HE, Stamler JS (2009). Thioredoxin-interacting protein (Txnip) is a feedback regulator of S-nitrosylation. J Biol Chem.

[50] Papadia S, Soriano FX, Leveille F, Martel MA, Dakin KA, Hansen HH, Kaindl A, Sifringer M, Fowler J, Stefovska V, Mckenzie G, Creaigon M, Corriveau R, Ghazal P, Horsburgh K, Yankner BA, Wyllie DA, Ikonomidou C, Hardingham GE (2008). Synaptic NMDA receptor activity boosts intrinsic antioxidant defences. Nat Neurosci.

[51] Wade MA, Tucker HN (1998). Antioxidant characteristics of L-histidine. J Nutr Biochem.

[52] Ralph DM, Robinson SR, Campbell MS, Bishop GM (2010). Histidine, cystine, glutamine, and threonine collectively protect astrocytes from the toxicity of zinc. Free Radic Biol Med.

[53] Webster KA, Prentice H, Bishopric NH (2001). Oxidation of zinc finger transcription factors: physiological consequences. Antioxid Redox Signal.

[54] Dinkova-Kostova AT, Holtzclaw WD, Wakabayashi N (2005). Keap1, the sensor for electrophiles and oxidants that regulates the phase 2 response, is a zinc metalloprotein. Biochemistry.

[55] Bragadin M, Scutari G, Folda A, Bindoli A, Rigobello MP (2004). Effect of metal complexes on thioredoxin reductase and the regulation of mitochondrial permeability conditions. Ann NY Acad Sci.

[56] Kruczek C, Görg B, Keitel V, Bidmon HJ, Schliess F, Häussinger D (2011). Ammonia increases nitric oxide, free Zn(2+), and metallothionein mRNA expression in cultured rat astrocytes. Biol Chem.

[57] Prakash R, Mullen KD (2010). Mechanisms, diagnosis and management of hepatic encephalopathy. Nat Rev Gastroenterol Hepatol.

